# Cancer research in the era of next-generation sequencing and big data calls for intelligent modeling

**DOI:** 10.1186/s40880-015-0008-8

**Published:** 2015-04-11

**Authors:** Jari Yli-Hietanen, Antti Ylipää, Olli Yli-Harja

**Affiliations:** Department of Signal Processing, Tampere University of Technology, P. O. Box 553, Tampere, 33101 Finland

**Keywords:** Cancer research, Big data, Mathematical modeling

## Abstract

We examine the role of big data and machine learning in cancer research. We describe an example in cancer research where gene-level data from The Cancer Genome Atlas (TCGA) consortium is interpreted using a pathway-level model. As the complexity of computational models increases, their sample requirements grow exponentially. This growth stems from the fact that the number of combinations of variables grows exponentially as the number of variables increases. Thus, a large sample size is needed. The number of variables in a computational model can be reduced by incorporating biological knowledge. One particularly successful way of doing this is by using available gene regulatory, signaling, metabolic, or context-specific pathway information. We conclude that the incorporation of existing biological knowledge is essential for the progress in using big data for cancer research.

## What is big data?

A report on new biology emphasizes the great potential of biosciences in the areas of health, food, energy, and environment in the 21st century [1]. The report predicts that global problems in these areas will be resolved through scientific integration. Furthermore, the report suggests that as biology develops into an information-based science, the key to successful scientific integration lies in the development of computational methods. These include support for data integration, model development for systems biology, and visualization of large masses of data. The development of these methods inevitably depends on systematic collection of large amounts of loosely connected, inherently noisy, and heterogeneous data, commonly called big data.

The volume, accumulation velocity, and complexity of biological data have grown beyond any feasible manual processing scenario, easily fulfilling all criteria for big data. This ubiquitous term is currently often used to vaguely describe almost any big data. However, the volume of the data should not be taken as the sole hallmark of big data. Modern computers can be scaled up fairly easily to automate the repeating tasks that are necessary for analyzing even the largest data collections, assuming that the structure of the data is known and sufficiently stable. Instead, the mere complexity of the data can make even moderate-sized data sets extremely challenging to analyze. For example, the human genome sequence, while quite large in size, could be easily processed computationally if its complexity allowed us to describe how exactly it needs to be analyzed. Furthermore, emerging new measurement technologies constantly feed the growth of complexity in biological data sets. For example, in the Cancer Genome Atlas (TCGA) project, the principal difficulty arises from the multitude of data types, not from the data volume *per se* [2].

## Black box approaches can make limited predictions using big data

Intelligent machine-learning and pattern recognition algorithms are needed to systematically extract information from big data. Sometimes the expectation is that algorithms more or less automatically bring forward interesting and important information out of a mass of unstructured data. However, the definitions of “interesting” and “important” are assessed on a subjective and context-dependent basis, and the computer can only recognize correlations between variables, and the meaning of these numbers is missing unless programmed explicitly. Therefore, such enthusiastic expectations are not well-founded.

Machine-learning systems, where the core of the system is deliberately detached from the reality, are called black-box approaches, and they underline the irrelevance of the deduction process. The focus of black-box approaches is correlation-based prediction. All forms of input data are equally important for this system, and the core of the system is inaccessible and uninterpretable for domain experts, such as oncologists. In a black-box approach, the contribution of the domain expert is usually focused on preprocessing data and selecting promising features for further processing.

For example, the self-organizing map (SOM) is an established black-box method of machine learning and visualization. Given the topology and convergence properties, SOM self-organizes and adapts to the input data. The entire process can be seen as execution of an optimization algorithm. It is sometimes claimed that SOM with an adaptive topology could reveal the secrets buried in the data. However, from the point of view of optimization, adaptive topology is equivalent to having more free parameters, thus adding to the problem instead of solving it. The same applies to the deep-learning approach. Unless real-life meaning is coded by manually preselecting the cost functions and the topology of the deep-learning method, the entire process is reduced into observing correlations between variables unattached from reality.

To conclude, black-box approaches can be used to make predictions by using big data. However, these approaches are limited in their capability to shed light beyond the observed data, namely gaining a system-level understanding of the mechanisms responsible for the data.

## The curse of dimensionality rules out black-box methods in cancer research

The availability of big data is sometimes expected to lead to fruitful results in data interpretation by using black-box methods. However, a leading figure of the machine-learning community warns against overeager expectations [3].

In cancer research, there is a genuine need to use the accumulated domain knowledge to cope with limited sample sizes and the ever-increasing data volumes. As the complexity of the black-box models, such as SOM or deep learning, increases, their sample requirements grow exponentially. This growth stems from the fact that the number of combinations of variables grows exponentially as the number of variables increases. Thus, a large sample size is needed to limit the probability of finding a fitting model by chance alone [3]. As a rule of thumb, thousands of samples facilitate the inferences made regarding a handful of variables in general-purpose black-box models. With millions of variables stemming from contemporary sequencing methods, even the size of the entire human population is dwarfed by the sample size requirements. However, in cancer research, the situation is typically such that the number of samples (usually number of patients) is relatively small although we have a large volume of data. Thus, it is necessary to limit the number of free variables that are inferred, leading us to consider a modeling approach that builds on the existing domain knowledge.

## Mathematical modeling is required for a deeper understanding

In contrast to black-box approaches, a mathematical model has better potential to integrate the existing domain knowledge to the desired extent. Mathematical models are often used as computational models—an obvious example being a mathematically defined group of differential equations for which solutions can be derived computationally through simulation. A mathematical model is used in science for a two-fold purpose: first, to understand and convey understanding of observed phenomena, and second, to predict the state of a system given initial conditions [4]. In addition to the prediction capability, the modeling approach facilitates an incremental understanding of the domain area through interpretation of the model parameters. Needless to say, black-box approaches are usually much more easily implemented for complex systems because very little domain knowledge can be implemented into them.

## Machine learning and mathematical modeling -- two sides of the same coin

If we accept the view that machine learning is a special case of mathematical modeling, both can be easily defined in similar terms. A machine-learning system includes an internal representation that directly corresponds to a mathematical model. The processes of machine learning and mathematical modeling include parameter fitting (technically, optimization). Finally, the goals of machine learning and mathematical modeling coincide. Both aim to predict outcome based on measurement data, e.g., patient survival may be predicted based on clinical observations. Just as importantly, the goal of machine learning and mathematical modeling is to gain a broad-level understanding of the subject area by observing how well different models fit the data. However, a mathematical model facilitates the accumulation of knowledge into its structure and parameter values. Instead, a black-box approach has a more predetermined structure, detached from reality, and its knowledge accumulation capacity is restricted to preprocessing of input data and preselection of promising features.

In the following section, we illustrate the differences between black-box and modeling approaches using an example from everyday life.

### Example 1. How can big data help fix a radio?

Let us trace the footsteps of the famous article “Can a biologist fix a radio?” [5]. Assume that we have a set of transistor radios of which some are broken. Our goal is to find out how to fix the broken radios using either a black-box or modeling approach. We base our understanding on the measurements of a few properties of the radios, for example, localized electric potential, temperature, visual integrity, and acoustic measurements. Even for a small set of radios, the volume of data can be overwhelming, e.g., measuring electric potential with a typical radio frequency of 1 GHz produces one billion samples per second. When sampling from multiple points and after adding other measurements, such as temperature, we can justifiably call our measurement set “big data.”

In the black-box approach, if we do not use any domain knowledge, we obtain correlations between the measurements and the statuses of the radios. One logical conclusion might be that heat is required for the radio to operate if working radios are slightly warmer than broken radios. It is easy to see that the black-box approach is not particularly efficient in learning how to fix the radios. For example, heating radios to the appropriate temperature does not fix them.

The modeling approach, on the other hand, permits domain experts to increase our knowledge on the radios. If we obtain help from electrical engineers, they will be able to improve the results by restricting the measurements to the sensible ones using their knowledge on radios, and perhaps they will even include the circuit diagram of these particular types of radios. We might be able to concentrate on the electric potential measurements of suspicious components to detect abnormalities that might result from a broken wire between the components or to isolate a faulty component that might cause the radio to appear broken.

## Integration of domain knowledge is required for machine learning in cancer research

In cancer research, the situation is typically such that we have a large volume of data, for example, from genome-wide expression, copy number, or methylation measurements, but the number of samples is relatively small. This situation prevents the use of many datum-mining algorithms, and we end up in a familiar situation, where genome-wide measurements result in the identification of one gene or of another feature, such as a mutation, instead of giving a deeper understanding of what is occurring in the gene regulatory network of cancerous cells. Thus, we argue that the inclusion of domain knowledge is necessary in limiting the degrees of freedom to a level where machine learning and big data can be fully used in cancer research.

One particularly successful way of introducing domain knowledge to genome-wide data is by using gene regulatory, signaling, metabolic, or context-specific pathways. These pathways contain information on genes, in relation to each other, that often work in concert towards a phenotypic action, such as cell movement, or something more intangible, such as growth signaling, giving the cell’s interaction a biological context. If we refer to the previous example, measurements of genes are much like measurements of the radio’s components or other properties. They are ambiguous without context and do not offer much help in understanding how the entire system works or why it does not work the way it should. Continuing with the analogy, pathways give genes a context whereby they influence the system, much like circuit diagrams show connections of the components in the radio, an inarguably valuable tool in understanding and fixing the device.

Pathway activities or inactivity are often more interesting than individual genes or mutations. This is not only because different aberrations can bring similar cancer phenotypes, for example the mouse double minute 2 homolog (MDM2)-p53 interaction, but also because pathways are closer to the phenotype that is often the context of interest in cancer research, such as “are the cells dividing after the treatment or not?” Computationally, the appealing feature in pathways is that they can reduce the dimensionality of gene expression data from tens of thousands of genes to hundreds of pathways. This reduction also makes genome-wide data much more digestible for researchers. Therefore, in many applications, researchers rather work on the pathway level than on the gene level, and many different pathway analyses have been implemented to date.

Recent genomic and molecular characterizations of cancer, especially the findings reported by the TCGA project, have shed light on cancer heterogeneity and potential targeted therapeutics, for example by recognizing new subtypes of gastric cancer [6]. In general, targeted computational methods, which make effective use of the available multimodal biological information, can significantly improve our ability to identify candidate biomarkers and targets and to conduct functional analyses [7]. For example, reducing the redundancy in enrichment analysis helps to reveal gene ontology modules efficiently and systematically [8].

### Example 2. Using pathway-level information connected with TCGA data

To become malignant cancer cells, normal cells must acquire a set of mutations that confer “hallmark” traits, such as increased proliferation, immortality, and invasiveness [9]. Usually, a single mutation is not enough to result in malignant growth, but several genes contributing to the process need to be “hit” before a pivotal phenotypic change takes place. Acquiring these traits can be conveniently described and understood as alterations in pathways. In many settings, cancers are classified based on the status of a single (often actionable) gene, such as *Her2* amplification-positive breast cancers and *KIT* mutation-positive gastrointestinal tumors, but almost all cancers have a characteristic set of somatic mutations that can be used to identify and classify the tumors and even to learn something about their clinical behavior.

After profiling a cohort of tumors, such as in the TCGA project, the common follow-up analysis is to cluster the tumors into subgroups based on genomic features, such as gene expression. Sometimes the projection of clinical data on top of the clusters reveals different clinical courses for the patients in each group, but more often, there is no clear difference in physiology. A similar deduction can also be conducted beginning from the gene expression that makes each group different, except that we do not currently understand the function of many genes, and even if we do, we do not know how to connect their molecular functions to the tumor phenotype. Furthermore, gene expression data can be quite different for many similar tumors because aberrations in different genes can cause similar phenotypic effects.

Statistical tools for investigating set enrichment can reveal hidden trends in gene lists that differ between tumor subgroups; for example, surprisingly, many radiation resistance-related genes differentiate one subgroup from others. The set enrichment tools also have the advantage of not being very sensitive to noisy gene expression data. Pathway-level results might hint as to why the clinical course for these patients might have been different than others. If enrichment analysis is performed systematically for hundreds of pathways, those data can be used to profile each subgroup. Alternatively, if we compute the enriched pathways for each sample prior to clustering, we can use the enrichment data to cluster tumors into subgroups that might be easier to interpret and understand. Pathway analysis offers intriguing opportunities; for example, if we know that the pathway activation profiles of two subgroups of different cancers are similar, we might hypothesize that both can be treated effectively by the same drug [10].

## Conclusions

The bigness of data does not come to the rescue, as the sample size requirements will always outgrow the number of available samples. Thus, the blind use of black-box methods will not be a working solution for analyzing big data in cancer research. The use of modeling approaches, which incorporate existing domain knowledge, is essential for the progress in this field.
